# Polyhexamethylene Biguanide Reduces High-Risk Human Papilloma Virus Viral Load in Cervical Cell Samples Derived from ThinPrep Pap Test

**DOI:** 10.3390/cimb46050293

**Published:** 2024-05-17

**Authors:** Ludovica Di Fraia, Carla Babalini, Marco Calcagno, Sara Proietti, Elisa Lepore, Pietro Di Fraia

**Affiliations:** 1 Di Fraia Laboratori s.r.l., 00034 Colleferro, Italy; 2 Department of Obstetrics and Gynecology, Santo Spirito Hospital, 00193 Rome, Italy; 3 R&D Department, Lo.Li. Pharma, 00156 Rome, Italy

**Keywords:** PHMB, HPV, viral load, cervical cell samples, ThinPrep Pap test

## Abstract

Human papilloma virus (HPV) infection and its progression still represent a great medical challenge worldwide. Clinical evidence has demonstrated the beneficial effects of polyhexamethylene biguanide (PHMB) on HPV clinical manifestations; however, evidence of the effect of this molecule on HPV viral load is still lacking. In this in vitro study, 13 ThinPrep Papanicolaou (Pap) tests were treated with a PHMB solution (0.10 g/100 mL) for 2 h. We observed no cytological changes but a significant reduction in the viral load of high-risk (HR) HPV after PHMB treatment, also revealing a dose-dependent antiviral effect. In addition, by stratifying the obtained results according to HR-HPV genotype, we observed a significant reduction in the viral load of HPV 16, P2 (56, 59, 66), 31, and P3 (35, 39, 68) and a strong decrease in the viral load of HPV 45, 52, and P1 (33, 58). Overall, 85% of the analyzed cervical cell samples exhibited an improvement in HPV viral load after PHMB exposure, while only 15% remain unchanged. For the first time, the data from this pilot study support the activity of PHMB on a specific phase of the HPV viral lifecycle, the one regarding the newly generated virions, reducing viral load and thus blocking the infection of other cervical cells.

## 1. Introduction

Human papilloma virus (HPV) infection is the most common sexually transmitted infection (STI) worldwide. It is estimated that over 80% of sexually active individuals will acquire this infection once in their life [1].

Skin-to-skin contact and mucosal contact are the eligible methods of HPV transmission. In particular, sexual contact is the main source of infection, starting with the entry of HPV into the epithelium through micro abrasions. From that moment, the lifecycle of HPV starts: late proteins L1/L2 are necessary for penetrating the basal cells, while early proteins E1/E2 are needed for viral DNA replication in the parabasal layer. Once the new virions are assembled, they are released into the environment from the upper layers. In this way, HPV has the capacity to form virions and become transmissible at some point in its natural lifecycle [2,3].

According to the oncogenic potential, all the papillomaviruses are classified into low risk (LR), which usually lead to benign lesions (such as skin or genital warts, condylomas, recurrent respiratory papillomatosis) or precancerous lesions [4], and high risk (HR), which may induce a malignant transformation of the epithelial and mucosal tissues, thus leading to the formation of precancerous lesions [5]. It is now well established that HR-HPVs (for instance, HPV 16 and HPV 18) are the cause of cervical cancer and are relevant factors for anogenital (anus, vulva, vagina, and penis) and head/neck cancers [6]. Even though lesions related to LR-HPV are not life-threatening, they may cause distress to patients, requiring expensive and painful interventions, often without a definitive solution.

According to the Bethesda System, premalignant changes represent histological abnormalities, ranging from atypical squamous cells of undetermined significance (ASCUS) and low-grade cervical dysplasia (LSIL/CIN1), which could progress to moderate dysplasia or severe dysplasia/carcinoma in situ (HSIL/CIN2 or CIN3) in the cervical region [7]. The cytological diagnosis of the lesions derives from the analysis of the results of the Papanicolaou (Pap) test, through which physicians collect scraped cervical cells from the patient and evaluate the cytological aspect or the presence of eventual abnormalities. Those diagnosed with HSIL are referred for further assessment with colposcopy and biopsy to prevent invasive tumoral progression, while, in clinical practice, those with LSIL are usually followed up to prevent the development of HSIL and progression toward tumoral onset [8]. In addition, especially in case of abnormal results from the Pap test, patients may undergo an HPV DNA genotyping test, through which physicians may identify and define the presence of HPV and, in particular, the genotype of the virus responsible for the infection.

Up to date, the majority of available treatments to counteract HPV infection (cytotoxic, excisional, physical, and immunomodulatory therapies) may reduce related symptoms but are relatively ineffective in decreasing the HPV viral load and/or in reducing the likelihood of disease transmission [9]. To date, some local solutions have been examined, but each one resulted in adverse effects. An in vitro study performed on HeLa HPV-positive cells investigated the effect of 5-aminolevulinic acid photodynamic therapy on reducing HPV viral load [10]. However, during the clinical use of such approach, some people reported negative side effects, including burning, itching, and excruciating discomfort [11]. Other topical treatments, such as podophyllotoxin and imiquimod, also produce undesirable reactions. The former—an isolated podophyllum plant extract—may cause local reactions and have negative impacts on the liver and neurological profile and cause other symptoms [12]. Imiquimod, an immunity modulator, may cause localized erosions, erythema, and burning as the most typical side effects [11]. The more invasive treatments usually performed (cryotherapy, surgical excision, electrosurgery) are associated with several adverse complications, including cervical stenosis, bleeding, and infection [13].

Since the time of exposure to HR-HPV is directly associated with the risk of developing cervical cancer, finding noninvasive therapeutic agents able to improve the viral clearance is therefore highly necessary.

Polyhexamethylene biguanide (PHMB) is a polybiguanide oligomer with antimicrobial activity, showing promising results in the management of HPV, as a topical agent against cutaneous-related manifestations. PHMB has been widely used as an antiseptic in different medical applications [14], as a component in swimming pool sanitizers, and as an antiseptic in contact lens solutions and wound disinfectants, as well as a preservative in cosmetics [15].

PHMB is well known for its rapid and broad-spectrum antimicrobial properties against bacteria and fungi, and particularly for its efficacy and absence of induced resistance compared to antibiotics [16,17]. Consisting of 10–12 monomers with a positive charge, it can attach to the pathogen membrane, which has a negative charge, and destroy it. This interaction increases the fluidity and permeability of the bacterial cell membrane and leads to the death of the pathogen itself [16]. In addition, once membrane permeability is altered, PHMB may also interact with the DNA molecule of pathogen, thus causing its degradation. Thanks to its mechanism of action, PHMB does not affect neutral phospholipids, resulting in low toxicity against human cells and supporting its safety for use in humans [14]. PHMB is highly tolerated on all mucous membranes, shows bactericidal activity at very low concentrations, and has a broad microbial spectrum. It is usually applied for the treatment of wounds [17] and bacterial vaginosis [18]; episodes of induced resistance have never been reported during PHMB treatment. Previous evidence from Punjataewakupt and colleagues produced some concerns about the safety of the clinical application of several antimicrobial agents, including PHMB for wound healing [19]. In particular, they reported adverse effects when PHMB was used on exposed bone or cartilage area. However, a following review report from the European Guidelines for Antiseptic Application in Wound Treatment highlighted the areas suitable for the application of PHMB, including the gynecological one. Furthermore, the authors included topical use in their discussion, explaining its safety for use compared to oral or respiratory administration, which may instead lead to adverse serious reactions [20].

The mechanism of action on which PHMB based its antimicrobial activity consists of interactions with cell membrane components, suggesting that such molecules might have also antiviral or virucidal activity. Indeed, several studies have reported the antiviral activity of PHMB and other biguanides, for instance, against human immunodeficiency virus (HIV) and herpes simplex virus (HSV) [21,22]. As proposed in a recent work, in which the authors described the antiviral activities of PHMB against different types of human viruses, its mechanism of action starts with electrostatic interactions with nonenveloped viruses. Subsequently, PHMB attaches to the virus through electrostatic forces and then forms aggregates through hydrophobic forces, thus disrupting the viral capsid [23].

Regarding PHMB and HPV, a previous study showed that in a sample of 100 patients diagnosed with HPV infection, the local use of a PHMB-based gynecological solution increased the spontaneous regression rate of the infection after 6 months of application [24]. In addition, local application of a cream based on PHMB was effective in the treatment of external condylomas, showing a good safety profile during a 16-week period of use [25]. However, no studies have investigated the effect of PHMB on HPV viral load in order to determine whether treatments based on this molecule can contribute to reduce the amount of the present HPV DNA.

Therefore, the aim of this pilot in vitro study was to verify, for the first time, the effect of PHMB on HR-HPV viral load. In particular, we investigated the effects of PHMB treatment on cervical cell samples derived from ThinPrep Pap (Hologic, Marlborough, MA, USA) tests that were fixed in the appropriate vial.

## 2. Materials and Methods

### 2.1. The ThinPrep^®^ Pap Test

For this pilot in vitro study, a total of 13 cervical scraped samples were collected with an endocervical brush and spatula (Cytobrush Plus GT and Pap Perfect Plastic Spatula; Medscand, Trumbull, CT, USA) and placed into a ThinPrep Pap test vial containing 20 mL of PreservCyt^®^ Solution (Cytyc Corp., Marlborough, MA, USA) to be analyzed. In this way, cervical cells were fixed in an alcohol-based solution constituting 35–55% methanol, without altering or disrupting permeability of such cervical cells. Cytological analysis and relative results were classified using the 2001 Bethesda System for reporting cervical cytology [26].

### 2.2. PHMB Solution and Treatment

Once each cervical cell sample was collected into a ThinPrep vial, it was divided in two aliquots in order to test the HPV viral load before and after treatment with PHMB: *(i)* a control aliquot was directly processed with BD Onclarity^TM^ HPV Assay(BD, Franklin Lakes, NJ, USA) (pretreatment condition); *(ii)* the other aliquot was exposed to 200 µL of a stock solution of PHMB (0.10 g/100 mL) for two hours at room temperature and then processed with a BD Onclarity^TM^ HPV Assay (post-treatment condition). In addition, a dilution of 1:50 from the stock solution of PHMB was tested by using a 24 h treatment in order to evaluate whether different concentrations of PHMB had different effects on HPV viral load in this setting of scraped cervical cells.

**Figure 1:**
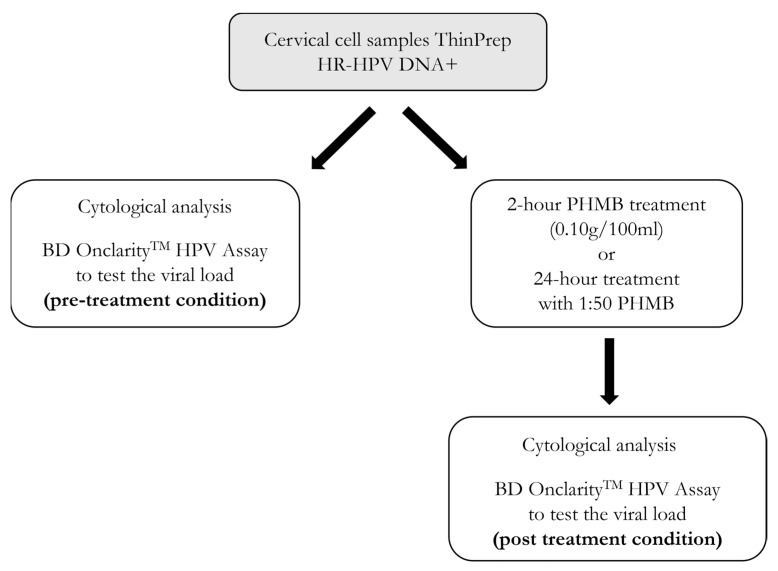
Flow chart of the experimental design of the in vitro study. Each sample of scraped cervical cells was put into a ThinPrep Pap test vial and divided into two aliquots to perform the cytological analysis and the analysis of the HPV viral load both before and after treatment with PHMB solution.

The cytological analysis was performed both in the pre- and post-treatment conditions. Figure 1 clearly depicts the experimental workflow of the in vitro study.

### 2.3. BD Onclarity^TM^ HPV Assay

The BD Onclarity^TM^ HPV Assay (BD, Franklin Lakes, NJ, USA) detects 14 HR-HPV genotypes, enabling extended genotyping through individual detection of HPV 31, 51, and 52 (in addition to 16, 18, and 45) and the pooled detection of 33/58 (P1), 56/59/66 (P2), and 35/39/68 (P3). An endogenous human beta-globin sequence is detected as a sample validity control, enabling sample extraction and showing amplification efficiency [27,28].

All samples were tested following the manufacturer’s instructions: briefly, 0.5 mL of each cervical sample was added to the LBC tube (a suitable solution produced by BD) to reach a final volume of 2.2 mL. From this solution, 0.8 mL of sample was automatically taken by the instrument to perform nucleic acid extraction using the extraction chemistry developed by BD (BD FOX™, Franklin Lakes, NJ, USA). The extracted DNA was then eluted to a final volume of 400 microliters, and 50 microliters was automatically pipetted into each of the three wells containing the dried master mix to perform real-time polymerase chain reaction (PCR). An algorithm verified the adequacy of the sample using the amplification of the human beta-globin gene.

### 2.4. Statistical Analysis

The relative DNA viral load of the HR-HPVs was calculated using the 2^^−^^ΔΔct^ method, as described by Livak and Schmittgen [29]. For this method, the cycle threshold (ct) values obtained from the BD Onclarity™ HPV assay were used and are reported as fold change to enhance the data’s intuitiveness and interpretability on a linear scale.

Data were analyzed using GraphPad software (version 8.0.1, La Jolla, CA, USA). The nonparametric Mann–Whitney U test was used for the analysis between pre- and post treatment conditions and the analysis between a single different genotype and the control condition. All descriptive parameters are stated as mean ± SEMs. A *p*-value of ≤0.05 was considered statistically significant.

## 3. Results

### 3.1. Characteristics of the Collected Samples

All the results of the cytologic analysis of the ThinPrep Pap tests are reported in Table 1. A total of eight samples were positive for LSIL (8/13; 61.5%); four samples were positive for ASCUS (4/13; 30.8%); one sample had no intraepithelial lesions or malignancy (NILM) but only bacterial vaginosis (BV) (1/13; 7.7%). No cytological changes were found either after 24 h of treatment with 1:50 diluted PHMB solution or after the exposure to 2 h of treatment with PHMB stock solution.

### 3.2. HR-HPV Prevalence and Genotype Distribution

The data on the genotype distribution among the collected cervical cell samples are reported in Table 2.

### 3.3. HPV Viral Load Detected through the BD Onclarity^TM^ HPV Assay

**Figure 2:**
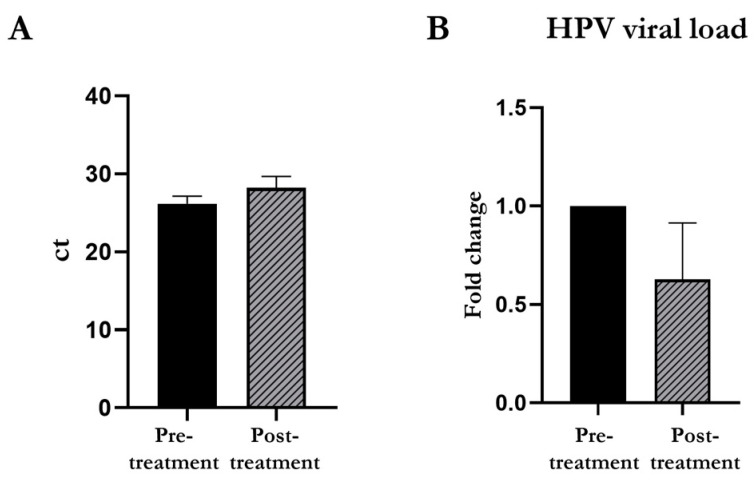
Effects of PHMB treatment (1:50 dilution) on HPV viral load in cervical cell samples. The 24-h treatment with 1:50 diluted PHMB solution induced a slight decrease in HPV viral load, evaluated both as ct (**A**) and as fold change (**B**), however, without reaching statistical significance.

We evaluated the effect of 1:50 diluted PHMB solution on the HPV viral load in the cervical cell samples on the ThinPrep Pap test. As reported in Figure 2, no significant changes were observed after 24 h of treatment with diluted PHMB. The detected ct values were slightly increased after treatment compared to the pre-treatment condition, without reaching statical significance (pre-treatment 26.18 ± 0.98 vs. post treatment 28.23 ± 1.45). In line with this, the fold change between ct values of the two conditions showed a slight improvement after PHMB treatment, however, without statistically significant variation (pre-treatment 1 vs. post treatment 0.63 ± 0.29).

**Figure 3:**
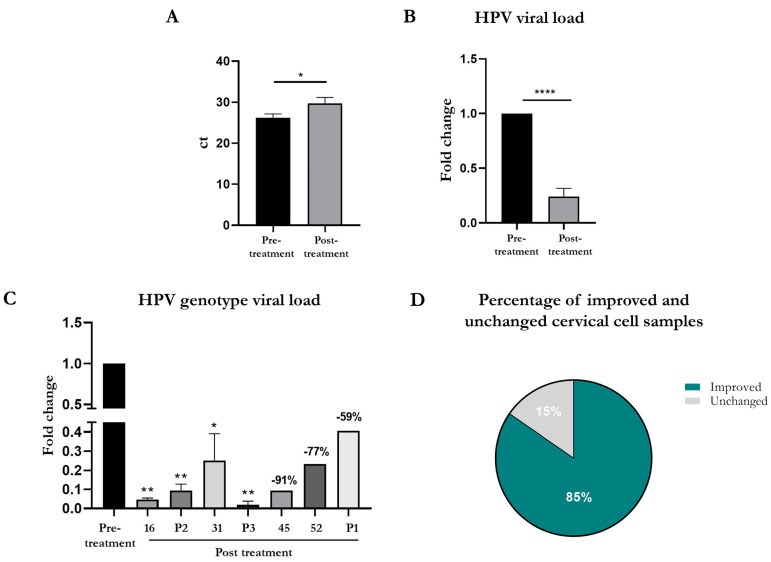
Effects of PHMB (stock solution) treatment on HPV viral load of scraped cervical cell samples. The 2-h treatment with PHMB (0.10 g/100 mL) significantly reduced HPV viral load, evaluated both as ct (**A**) and fold change (**B**). In addition, by dividing samples according to HR-HPV genotype (**C**), PHMB significantly reduced viral load of HPV-16, P2, P3, and 31 and strongly reduced viral load of HPV 45, 52, and P1 compared to pretreatment condition. Overall, by evaluating the percentage of improved and unchanged cervical cell samples (**D**) in terms of viral load, the HPV viral load was reduced in 85% of the analyzed Thin Prep Pap test samples after exposure to PHMB solution (nonparametric Mann–Whitney U test, * *p*-value < 0.05; ** *p*-value < 0.01; **** *p*-value < 0.0001).

Subsequently, we tested the effects of stock PHMB solution (0.10 g/100 mL) on HPV viral load. We observed a significant increase in detected ct values (Figure 3A) in the post-treatment condition compared to the pretreatment condition (pretreatment 26.18 ± 0.98 vs. post treatment 29.75 ± 1.43, *p*-value = 0.0372), thus reflecting a reduction in HR-HPV viral load after 2 h of exposure of cervical cell samples to PHMB. In addition, the analysis of the fold change between ct values of the two conditions confirmed the significant reduction (pre-treatment 1 vs. post treatment 0.24 ± 0.08, *p*-value < 0.0001) in the cervical cell samples treated with PHMB (Figure 3B).

Furthermore, we analyzed the data from the stock PHMB solution by stratifying the results according to the different HR-HPV genotypes. As reported in Figure 3C, the viral load for most of the analyzed genotypes was significantly reduced after PHMB treatment. After 2 h of PHMB treatment, the viral load was strongly and significantly reduced compared to the control condition for HPV-16 (1 vs. 0.05 ± 0.007, *p*-value < 0.01), P2 (including HR-HPV 56, 59, 66; 1 vs. 0.09 ± 0.03, *p*-value < 0.01), P3 (including HR-HPV 35, 39, 68; 1 vs. 0.02 ± 0.02, *p*-value < 0.01), and HPV-31 (1 vs. 0.25 ± 0.14, *p*-value ≤ 0.05).

The remaining genotypes—HR-HPV 45, 52, and P1 (including HR-HPV 33, 58)—even though they did not reach statistical significance due to the low number of the available samples, reductions of 91%, 77%, and 59% were achieved after PHMB treatment, respectively.

Overall, regarding the percentage of cervical cell samples that showed improved or unchanged viral load after PHMB treatment (Figure 3D), we observed that the HPV viral load was reduced for 85% of the analyzed ThinPrep Pap tests after 2 h of PHMB treatment, compared to 15% that remain unchanged.

## 4. Discussion

In this experimental in vitro pilot study, we demonstrated that PHMB may reduce the HPV viral load in scraped cervical cell samples placed into appropriate ThinPrep Pap test vials. In addition, we found that the observed effect against HPV viral load seemed to be dependent on PHMB concentration: indeed, the positive effects were clearly improved by using a more concentrated PHMB solution instead of its dilution (1:50). In the experiment with the diluted PHMB solution, we observed a trend of a reduction in HPV DNA viral load; instead, for the PHMB stock solution, the obtained data reached statistical significance.

Considering the starting experimental conditions of the ThinPrep Pap test, we did not expect changes in the cytological analyses of the lesions after PHMB treatment. In fact, when scraped cervical cells are inserted into the appropriate ThinPrep vial, they are exposed to Preserv Cyt, which is an alcohol-based fixative containing 35–55% methanol that maintains the cell’s morphology and DNA/RNA integrity for long periods, as experimentally evidenced [26]. Therefore, not observing cytological changes in the analyzed cervical samples after 24 h of treatment with diluted solution or after 2 h of exposure to PHMB stock solution could have been due to this starting experimental setting.

**Figure 4:**
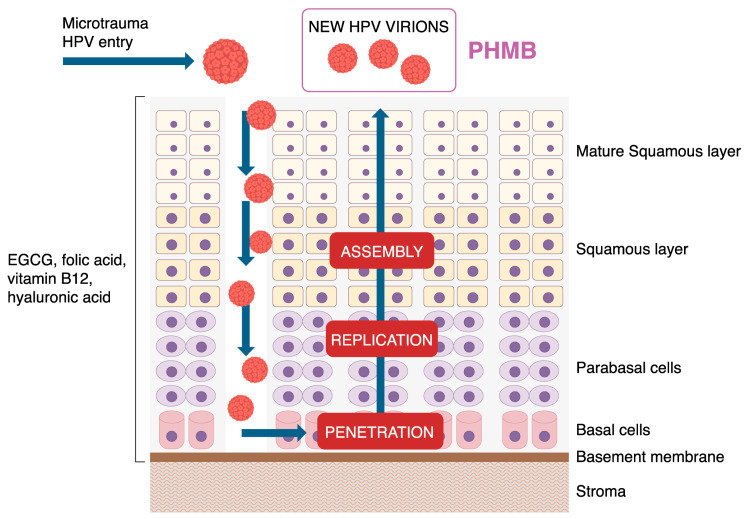
Proposed mechanisms of action of PHMB treatment on HPV viral particles. Our data support the mechanism of action of PHMB against a specific phase of the HPV life cycle, as shown in this figure. Considering the experimental conditions, this molecule seems to be able to act on the newly generated virions outside of the cervical cells, thus reducing the detected viral load and blocking the infection of other cells. These data allow us to consider the use of PHMB alone or in combination with natural molecules that may act on other phases of the viral lifecycle, such as virus entry, replication and penetration, as also reported in the review by Laganà and colleagues [30].

Furthermore, considering that the cervical cells in the ThinPrep Pap test are fixed and not lysed, it is reasonable to assume that PHMB does not act on DNA inside the cells, but it may act only on the DNA molecules that are present outside of the collected cervical cells, thus preventing the eventual effect of PHMB on the host’s intracellular DNA. In addition, the cellular lysis in DNA extraction takes place directly inside the BD Viper LT Instrument, according to the manufacturer’s manual; so, it occurs later than the timeframe of PHMB activity. For this reason, with fixed cervical cells, conceiving that PHMB acts on the DNA of viral particles outside of the collected cervical cells is a reasonable explanation for its mechanism of action. This is also consistent with the virus’s life cycle: HPV enters the epithelium through microabrasions; penetrates into basal cells thanks to late proteins L1/L2; and, at the parabasal layers, the early proteins E1/E2 allow viral DNA replication. Following assembly in the squamous layers, new virions are released into the upper layers are shed [2]. Overall, PHMB activity is directed against such newly generated HPV virions that could then infect other cells and perpetuate the viral infection. Figure 4 clearly describes the activity of PHMB according to the proposed model.

PHMB is an antimicrobial agent, basing its activity on electrostatic interactions. It is able to interact with a microorganism’s membrane, altering its permeability and degrading its DNA molecule. In addition, thanks to its mechanism of action, it preferably targets microbial cells versus mammalian cells, thus ensuring its safe use. Moreover, PHMB exhibits a favorable safety profile, in particular when compared to that of other commonly used antimicrobial and antiseptic agents [31,32].

Some scientific evidence also shows that its antiviral activity is attributable to its interactions with components of the virion membrane, as is the case with HIV and HSV [21,22]. In the case of HPV, a clinical study reported its beneficial effects against the clinical manifestations of the infection; however, in vitro studies highlighting the possible mechanism of action of PHMB against this virus are still lacking. A clinical study demonstrated the positive effect of PHMB on medium-grade HPV lesions or genital warts. After three months, the use of a PHMB-based gynecological solution induced a regression rate of the infection in 66% (33/50) of the patients compared to 56% (28/50) of the patients in the control group [24]. After six months of treatment, a significant difference in the regression rate was evident between the PHMB treatment and control groups (90% vs. 70%, *p* value= 0.023), without side effects related to long-term use being reported. Another clinical study demonstrated the useful application of PHMB for the treatment of HPV genital warts. Daily application of a PHMB cream for up to 16 weeks significantly cleared genital warts (52%) as compared to those of patients in the placebo group (4%, *p* value < 0.0001) [25]. These results support the topical use of PHMB against these clinical manifestations of HPV infection. These latter may occur in the anal or genital area or on the internal surface of the vagina and anus, and they may cause itching, redness, discomfort, and psychological distress. Topical agents, compared to ablative treatments (vaporization, resection, coagulation, or excision), may be very effective in treating such conditions, since they are self-applied by patients and are less traumatic than surgical intervention. However, patient compliance is inconsistent, and recurrences are highly common [33].

We are aware of the preliminary nature of this in vitro study and the small number of evaluated samples. Therefore, the findings of this study should be interpreted in the context of its limitations. For this reason, further tests and analyses on larger sample are necessary to support and expand our results.

Nevertheless, the obtained evidence on the effect of PHMB against HPV viral load may support the local use of this molecule as a safe and well-tolerated approach, effective in counteracting new HPV virions that could infect other cervical cells and perpetuate the infection.

Interestingly, this approach could be applied in association with other natural molecules whose oral administration has shown beneficial effects against HPV infection, targeting its persistence. Indeed, a recent study described the positive and antiviral effects of orally administered natural molecules such as epigallocatechin gallate (EGCG), folic acid (FA), vitamin B12, and hyaluronic acid (HA) [30], which are effective and safe in the management of HPV infection [34,35]. These molecules can counteract HPV persistence, replication, and penetration, improving the related cytology. Adding PHMB as a local treatment might help to act against another phase of the viral lifecycle by counteracting the newly generated virions outside of the cervical cells, arresting the perpetuation of the infection to the other cervical cells. Of course, as previously stated, additional studies are needed to corroborate this preliminary evidence.

## 5. Conclusions

HPV infection and treatment still represent a great medical challenge worldwide. Indeed, it is the most common sexually transmitted infection, but the available treatments are still ineffective in decreasing HPV viral load and/or reducing the likelihood of disease transmission. In this preliminary work, for the first time, a treatment with PHMB exhibited dose-dependent antiviral effects on HR-HPV-positive scraped cervical cell samples, demonstrated as a reduced HPV viral load after 2 h of PHMB treatment. Interestingly, the used experimental conditions highlighted the activity of PHMB toward a specific phase of the HPV lifecycle: the one regarding the newly generated virions outside cervical cells. As such, local treatment with PHMB may help to counteract the infection of other cervical cells by the newly generated viral particles, thus contributing to reducing the viral load and slowing the progression of the disease.

## Figures and Tables

**Table 1:** Results of the cytologic analysis of ThinPrep Pap tests. Low-grade intraepithelial lesion (LSIL); atypical squamous cells of undetermined significance (ASCUS); negative for intraepithelial lesion or malignancy (NILM); bacterial vaginosis (BV).

ThinPrep PAP Test Cytological Results	No. Samples = 13	%
LSIL	8	61.5%
ASCUS	4	30.8%
NILM (BV)	1	7.7%

**Table 2:** HR-HPV genotype distribution among cervical cell samples. Multiple HPV infections indicates at least 2 HPV genotypes found in the same sample. The detections of P1 (33, 58), P2 (56, 59, 66), and P3 (35, 39, 68) are categorized as part of the multiple HPV infections.

HR-HPV Genotype	No. Samples = 13	%
HPV 16	2	15.4%
HPV 31	4	30.8%
HPV 45	1	7.7%
HPV 52	1	7.7%
Multiple HPV infections	10	76.9%

## Data Availability

Data are available upon request from the corresponding author.
